# Gestational and Lactational Co-Exposure to DEHP and BPA Impairs Hepatic Function via PI3K/AKT/FOXO1 Pathway in Offspring

**DOI:** 10.3390/toxics11030216

**Published:** 2023-02-24

**Authors:** Minghan Wang, Yu Wang, Junyuan Han, Zhiwen Duan, Jiye Yin, Rigao Ding, Quanjun Wang

**Affiliations:** 1 State key Laboratory of Toxicology and Medical Countermeasures, Beijing Institute of Pharmacology and Toxicology, Beijing 100089, China; 2 Faculty of Life Sciences, China Medical University, Shenyang 110031, China; 3 Shenyang Medical College, School of Public Health, Shenyang 110121, China

**Keywords:** di-(2-ethylhexyl) phthalate, bisphenol A, mixture toxicity, metabolomics, PI3K/AKT/FOXO1 signaling pathway, molecular docking

## Abstract

Di-(2-Ethylhexyl) phthalate (DEHP) and bisphenol A (BPA) present significant environmental endocrine-disrupting chemical properties. Although studies have implied reproductive impairment from exposure to BPA and DEHP, no study to date has shown the effect and mechanism of hepatic function after gestational and lactational co-exposure to DEHP and BPA in offspring. A total of 36 perinatal rats were randomly divided into four groups, DEHP (600 mg/kg/day), BPA (80 mg/kg/day), DEHP combined with BPA (600 mg/kg/day + 80 mg/kg/day), and control. Notably, 11 chemical targets were screened after identifying eight substances associated with chemically-induced hepatic damage. Molecular docking simulations revealed a high-scoring combination of eight metabolic components and targets of the PI3K/AKT/FOXO1 signaling pathway. The DEHP and BPA combination disrupted hepatic steatosis, ultimately affecting systemic the glucose and the lipid metabolic homeostasis with significant toxicity. Mechanistically, co-exposure to DEHP and BPA causes liver dysfunction and hepatic insulin resistance via PI3K/AKT/FOXO1 pathway in offspring. This is the first study of the hepatic function and mechanism of co-exposure to DEHP and BPA that combines metabolomics, molecular docking, and traditional toxicity assessment methods.

## 1. Introduction

The high abundance of di-(2-Ethylhexyl) phthalate DEHP and bisphenol A (BPA) means that humans are regularly exposed to these substances, as they are ubiquitous and escaping them is effectively impossible. The mixture toxicity of these environmental endocrine-disrupting chemicals (EDCs) contributes significantly to disparities in metabolic disease risk that makes their study particularly interesting. Only 5% of all toxicological research has been currently focused on mixed chemicals [1,2,3,4,5,6,7,8,9,10,11,12]. While the united effects of EDC from the same category (e.g., estrogenic, thyroid-disrupting agents, or antiandrogenic) can be assessed using dose addition, EDC acts using alternative mechanisms [13]. An additional unfathomed issue is the toxicity of EDC mixtures. Increasing evidence shows that EDCs with similar modes of action (MoAs) can produce significant effects in an additive manner [14]. Furthermore, additional studies on different experimental models have revealed interaction potential among chemicals such as DEHP, BPA, and CYP, suggesting a potential synergism in their endocrine actions [15,16,17]. It is thus essential to use real-life risk simulation (RLRS) to determine the effect of the combined effects of such chemicals [18]. Previous studies have presented specific effects of DEHP and BPA on reproductive impairment. Nevertheless, the other specific effects and underlying mechanism of co-exposure to DEHP and BPA have not been described. Moreover, the current study, that is centered on the toxic effects of the evaluated chemicals, is mainly based on traditional animal or cell models that are commonly used. In the present study, gas chromatography coupled with mass spectrometry and liquid chromatography coupled with tandem mass spectrometry (LC-MS/MS) platforms (for monitoring polar and non-polar metabolites) were employed to delineate the alteration in metabolomics characteristics [19,20], while pathway analysis was employed to examine causal relationships and biomarkers.

## 2. Materials and Methods

### 2.1. Animal Feeding

A total of 90 specific pathogen-free, healthy Sprague Dawley rats (30 male rats and 60 female rats) (Liaoning Changsheng Biotechnology Co., Ltd. Shenyang, China, experiment Animal Production License No. SCXK (Liao) 2015-0001) were selected, weighing 150–200 g, and 10 weeks old. Animals were housed for one week before being entered into the study. The breeding environment complied with the GB14925-2010 ‘Experimental Animal Environment and Facilities’ 12 h/12 h day/night light cycle, temperature (25 ± 2 °C), and relative humidity (50 ± 2%). All of the rats were provided food (Changsheng Bio-Technology, Shenyang, China) and tap water libitum, and their body weights and organ weights were recorded. Animal protocols were approved by the Experimental Animal Ethics Committee of Shenyang Medical College Number: 20150609-GWDL, and conducted in compliance with the National Institutes of Health Guide for Care and Use of Laboratory Animals.

### 2.2. Establishment of Animal Models

The female rats were disposed with vaginal smear examination from 8:00–9:30, until sperm or vaginal plug was detected. Gestation Day 0 (GD0) was defined as that day. A total of 48 pregnant female rats were randomly assigned into four groups (*n* = 6), as follows. Control: coin oil; BPA: 80 mg/kg/day; DEHP: 600 mg/kg/day; BPA+DEHP: BPA 80 mg/kg/day and DEHP 600 mg/kg/day. The DEHP (600 mg/kg) was equal to 1/40 of the half lethal dose of DEHP for rats, and was also based on the no-observed-adverse-effect level (NOAEL) of 30 mg/kg body weight/day in rats [21]. Similarly, the BPA (80 mg/kg) was equal to 1/40 of the half lethal dose of BPA for rats, and also based on NOAEL of 5 mg/kg body weight/day in rats [22]. All rats were administered during gestation with 5 mL/kg of the relevant scheme by oral gavage from the 5th day of conception to the 21st day after delivery, while an equal volume of corn oil was given to the control group daily. On PND7, PND21 and PND56, rats were sacrificed randomly, with a total of six (three male and three female) in each group. The total number of progeny, the number of live progeny, and gender of the offspring were recorded as shown in Table 1, following the OECD 421/422 [23]. 

### 2.3. Blood Collection

Animals were euthanized under light intraperitoneal ketamine (75 mg/kg b.w.)/xylazine (10 mg/kg b.w.) injection of anesthesia at the endpoint of the experiment. Blood samples were collected by cardiac puncture after anesthetic administration. Serum was separated by centrifugation at 3000 r/min for 6 min and frozen (−20 °C) for biochemical analysis and detection of DEHP and BPA metabolites.

### 2.4. Organ Collection

Liver was collected and immediately washed in 0.9% saline and weighed to measure the relative organ weight ((ROW) = organ weight (g)/final body weight of the animal (g) × 100%), then packed in a collection bag and stored at −80 °C if required. Other liver samples were fixed in 10% formalin, embedded into paraffin and sectioned for morphological examinations. 

### 2.5. Biochemical Analysis

Biochemical parameters were detected by the corresponding test kits. All biochemical assays were tested with commercial reagents following good laboratory practice.

### 2.6. Real-Time Polymerase Chain Reaction (PCR) 

Real-time PCR was used to measure mRNA expression. Total RNA was extracted from liver islets using Trizol reagent. We performed cDNA synthesis and PCR reaction according to the reagent instructions. Then use “no amplification controls” (nac) and “no template controls” (ntc) for quality assessment in each PCR assay. The lists of primer sequences are presented in Table 2.

### 2.7. Western Blot Analysis 

The total protein of the samples was separated by SDS-PAGE and PVDF membranes. The membranes were blocked with TBS Tween 20 (0.075%) that contained 5% skim milk for 1 h. Then, PVDF membranes were incubated with targeted primary antibodies at 4 °C overnight. The antibodies against IRS2 (ab134101, Abcam, Cambridge, UK), p-IRS2 (ab178703, Abcam, Cambridge, UK), PI3K (ab302958, Abcam, Cambridge, UK), p-PI3K (CST#173866, Cell Signaling Technology, Danvers, DE, USA), p-AKT (ab38449, Abcam, Cambridge, UK), and GAPDH (CST#5174, Cell Signaling Technology, Danvers, DE, USA) were diluted to 1:1000 and FOXO1 (ab8805, Abcam, Cambridge, UK) antibodies were diluted to 1:500. Membranes were then washed with TBS Tween 20 (0.075%) three times in the following day, then incubated with horseradish peroxidase antibody (1:10,000) for 1 h and the membranes washed another three times with TBS Tween 20 (0.075%). The protein bands were monitored by development with high-sensitivity luminescent liquid (ECL). ImageJ software 4.0 (San Diego, CA, USA) was used to analyse the densitometry of specific bands.

### 2.8. Standard Solution Preparation 

A total of 5 mg of BPA was dissolved in methanol accurately. The volume was adjusted to 5 mL in a 5 mL volumetric flask, and the solution was sonicated for 30 min to prepare a standard solution of 1 mg/mL. A total of 5 μL DEHP and 5 μL MEHP were dissolved in 5 mL methanol using a 5 mL volumetric flask, and this was then sonicated for 30 min to prepare a standard stock solution of 1 mg/mL that was then stored at 4 °C.

### 2.9. Sample Preparation 

A total of 200 μL of serum was added in a stoppered glass tube, and 600 μL of acetonitrile was added and vortexed for 3 min. The supernatants were then transferred into new tubes after concentration using high-purity dried nitrogen at 37 °C, centrifuged at 4000 r/min for 10 min, then stored at –20 °C. A total of 200 μL of 70% acetonitrile in water was used for reconstitution, vortexed for 1 min, and centrifuged at 13,000 r/min for 10 min. A total of 100 μL of the supernatant was finally transferred to the injection vial.

### 2.10. UHPLC Conditions 

Mobile phase A: acetonitrile, mobile phase B: water, gradient elution: flow rate: 0.3 mL/min, injection volume: 10 μL. Table 3 summarizes the gradient elution conditions.

### 2.11. Mass Spectrometry Conditions 

A multiple reaction monitoring method was used for detection. The conditions that followed were as follows. Acquisition time: 0–17 min; scanning range: 100–1200 Da; scanning time: 0.5 s; collision energy: low energy 6 V, high energy 20–30 V.

### 2.12. Qualitative Method 

The method of combining the nuclear/mass ratio of chemical substances with the retention time of standard substances was used to identify the substances. Instrument variability was 4% for internal standards and total process variability for endogenous metabolites was 12%. The typical mass error was less than 5 ppm. Identification of known chemical entities was based on contrast of metabolomic library entries of purified standards.

### 2.13. Bodyweight

Bodyweight was measured daily before inoculation during the exposure period.

### 2.14. Oxidation Index 

A total of 50 mg of hepatic tissue was weighed into a sterile EP tube with 1.5 mL precooled saline. An amount of 10% of hepatic tissue was fully grinded (1500 r/min, 2 min) in a high-throughput tissue crusher (Beijing Dinghaoyuan Technology Co., Ltd. Beijing, China). The supernatant was then collected using a low-temperature high-speed centrifuge (Sigma, San Diego, CA, USA) at 4000 r/min for 10 min. Relevant indexes were measured following the kit instructions. The protein concentration of tissue homogenate was confirmed by the standard BCA method.

### 2.15. Histopathological Analysis 

Liver samples were collected from two offspring rats in all four groups randomly, preserved in 4% paraformaldehyde, dehydrated using a gradient of alcohol concentrations, cleavaged in paraffin in 5 mm-thick slices, and stained with hematoxylin and eosin for conventional morphological evaluation. Histological morphological assessments were performed using a light microscope (Olympus, Tokyo, Japan).

### 2.16. Collation and Network Construction of Liver Injury Related Targets 

Non-alcoholic fatty liver (NAFLD) was chosen as the keyword to obtain the relevant targets from the following databases: TTD (http://bidd.nus.edu.sg/BIDD-Databases/TTD/TTD.asp, accessed on 7 January 2022), Drug Bank (http://www.drugbank.ca/, version 4.3, accessed on 7 January 2022), GAD (https://geneticas-sociationdb.nih.gov/, accessed on 7 January 2022), and DisGeNET (http://www. disgenet.org/web/DisGeNET/menu, accessed on 7 January 2022). Cytoscape is a bioinformatics analysis software. Cytoscape3.6.0 (Cytoscape, San Diego, CA, USA).was then used to construct the disease-target network diagram and a visual molecular interaction network. In this software, each node (Node) is a gene, protein or molecule, and the node-to-node connection (Edge) represents the interaction between these biological molecules (https://cytoscape.org, accessed on 7 January 2022). All databsses were accessed on 7 January 2022.

### 2.17. Target Prediction for BPA, DEHP, and Their Metabolites 

The related targets of BPA, DEHP and metabolites were obtained from the PubChem database (https://pubchem.ncbi.nlm.nih.gov/, accessed on 7 January 2022) and the Swiss Target Prediction website (http://www.swisstargetprediction.ch/, accessed on 7 January 2022). Cytoscape3.6.0 was then used to construct the metabolite-target network diagram. These databsses were accessed on 12 December 2021.

### 2.18. Network Construction and Analysis 

Network construction was carried out by the merged function of Cytoscape (ver.3.6.0) to discover the common targets (metabolic targets) by merging the disease-target network diagram and metabolites-target network diagram.

### 2.19. Gene Ontology and KEGG Pathway Enrichment Analysis 

To investigate the toxicity mechanism of the evaluated chemical substances, Gene ontology KEGG and Metascape (http://metascape.org/, accessed on 12 January 2022) were used to analyze pathway enrichment of the predicting targets. The critical value of significant function and pathway was *p* < 0.5.

### 2.20. Molecular Docking 

To better understand the mechanism of hepatic glucose metabolism, computer-aided molecular docking (MOE) was conducted to simulate the binding of DEHP and BPA prototypes and metabolites with proteins in the PI3K/AKT signaling pathway. CB-Dock (http://cao.labshare.cn/cb-dock/, accessed on 12 January 2022) is a protein-ligand docking method that identifies binding sites of a specific protein and calculates the centers and sizes with a novel curvature-based cavity detection approach that can enhance the hit ratio and accuracy of blind docking. The mol2 structure of the compounds was obtained from the TCMSP database. We used the PDB database (https://www.rcsb.org/, accessed on 12 January 2022) to obtain the 3D structures of the target proteins. Then we uploaded the 3D structure of the target protein and the mol2 structure of the compound using the CB-Dock webserver.

### 2.21. Statistical Analysis 

Statistical analyses of the gene expression data were performed by the GraphPad Prism 6.0 software (GraphPad Software. Inc., San Diego, CA, USA) and SPSS 22.0 software (SPSS. Inc., San Diego, CA, USA). All the data are expressed as mean ± standard error of mean (SEM). The measurement data are subjected to normality test, following normal distribution, or approximate normal distribution. One-way ANOVA was used to compare the mean between groups, followed by the Student-Newman–Keuls test for multiple post hoc comparison tests. The LSD test is used when comparing the variances of the two pairs, and the Dunnett T3 test is used when the variance is not uniform. The relationship between the indicators was analyzed using the factorial design analysis of variance. The significance level was defined as *p* < 0.05 in all tests.

## 3. Results

### 3.1. Identification and Qualitative Analysis of BPA, DEHP, and Their Metabolites in the Blood of Gestational Rats 

**Figure 1:**
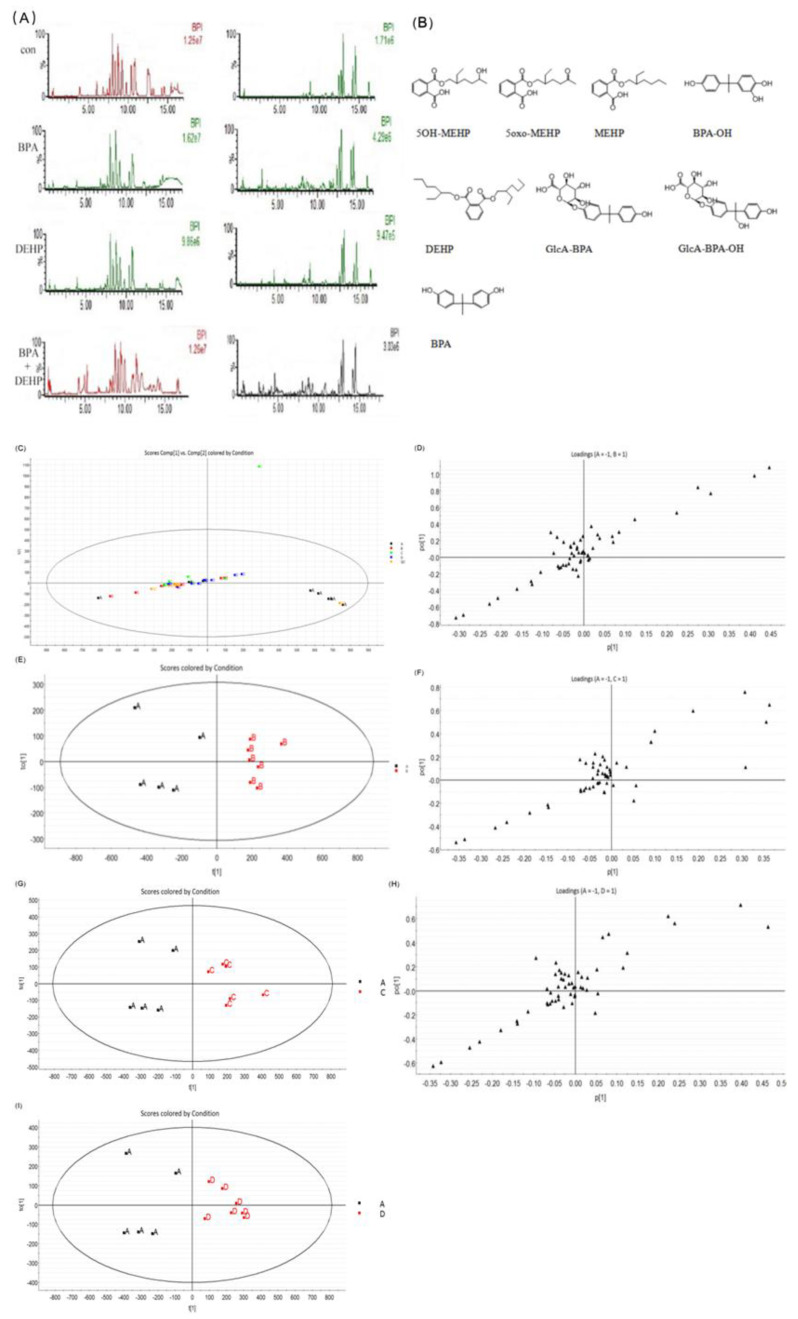
BPI chromatogram of blood samples from each group in positive (left) and negative (right) ion modes (**A**). Chemical structure of BPA, DEHP and its metabolites (**B**). The plasma principal component analysis score of the mother between the control group and the exposed group. Vector, loading map (**C**–**I**). Data are presented as mean ± SEM. [Note]: A: corn oil group, B: BPA group, C: DEHP group, D: BPA+DEHP group. Each represents a marker.

The retention time and the characteristic fragment ions of BPA, DEHP, and their metabolites in plasma are presented in Table 4 under the specified UPLC- Q-TOF/MS conditions. We used the blood of gestational rats for analysis. The total ion chromatogram components of the control, BPA, DEHP and BPA+DEHP groups are presented in Figure 1A. The chemical structures of BPA, DEHP and their metabolites are demonstrated in Figure 1B. The chromatographic peaks were identified in negative ion scanning mode at 0–11 min, while the combination group presented the most abundant chromatogram. The original plasma samples of MarkerLynx software were used for principal component analysis (PCA) in order to identify the difference of plasma metabolite profiles between the control and tested groups. The score vector (score plot) and the loading map were analyzed using orthogonal least squares discriminant (OPLS-DA) (Figure 1C–I). The potential markers from the corresponding loading map were identified after grouping the score graph. Significant changes were demonstrated in the direction of Component 1, which contributed the most for grouping (*p* < 0.05).

### 3.2. Effects of BPA and DEHP Mixed Exposure on the Reproductive Development of Offspring

**Figure 2:**
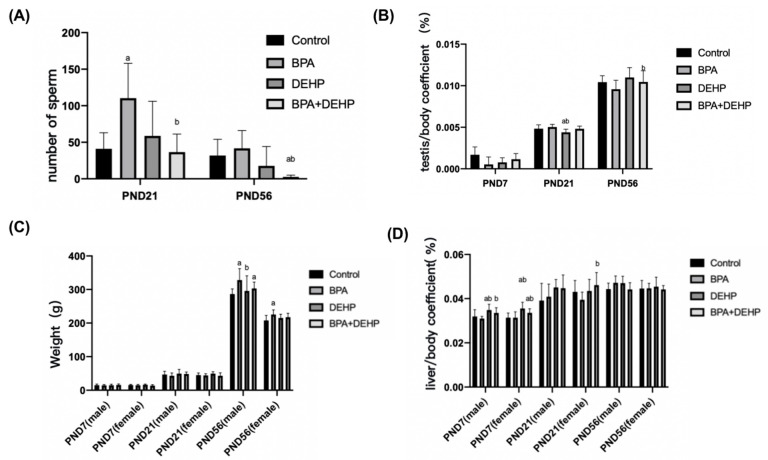
Number of sperm on PND7, PND21 and PND56 of offspring (**A**). Tesis/body coefficient of offspring on PND7, PND21 and PND56 (**B**). Weight changes of male and female offspring exposed to BPA, DEHP and BPA+DEHP on PND7, PND21 and PND56 (**C**). H_2_O_2_ assay of offspring exposed to BPA, DEHP and BPA+DEHP. Relative liver weight of offspring in male and female exposed to BPA, DEHP and BPA+DEHP on PND7, PND21 and PND56 (**D**). Data are presented as means ± SEM. [Note]: “a”: compared with control, “b”: compared with BPA group, *p* < 0.05, *n* = 6.

In the BPA+DEHP group, there was a significant decrease in sperm counts in comparison with the control group on PND56 (Figure 2A, *p* < 0.05). On PND21, a significant decrease in the number of sperm compared to the control group was observed in the BPA group (Figure 2A, *p* < 0.05). In the BPA+DEHP group, there was a significant decrease of the tesis/body coefficient compared to the BPA group on PND56 (*p* < 0.05). The tesis/body coefficient was also decreased significantly in the DEHP group compared to the control group on PND21 (Figure 2B *p* < 0.05). The body weight was significantly increased in the BPA and DEHP groups on PND56 in males (*p* < 0.05), while there was no significant change only in the BPA group on PND21. Moreover, there was a significant decrease in the DEHP group compared to the BPA group on PND56 in females, indicating a sex-specific difference (Figure 2C, *p* < 0.05). The liver/body coefficient of DEHP and BPA+DEHP groups was obviously higher compared to the control group, so as the BPA+DEHP group compared to the BPA group on PND7 in males and females (Figure 2C, *p* < 0.05). A significant increase in the liver/body coefficient was observed in the BPA+DEHP group compared to the BPA group of females on PND7 (*p* < 0.05), whereas no obvious change was observed in all groups on PND21 (Figure 2D, *p* < 0.05).

### 3.3. Effects of BPA and DEHP Mixed Exposure on Hepatic Oxidative Stress of Offspring

#### 3.3.1. Histological Analysis of the Hepatic Sections Stained with Haematoxylin and Eosin in Offspring

**Figure 3:**
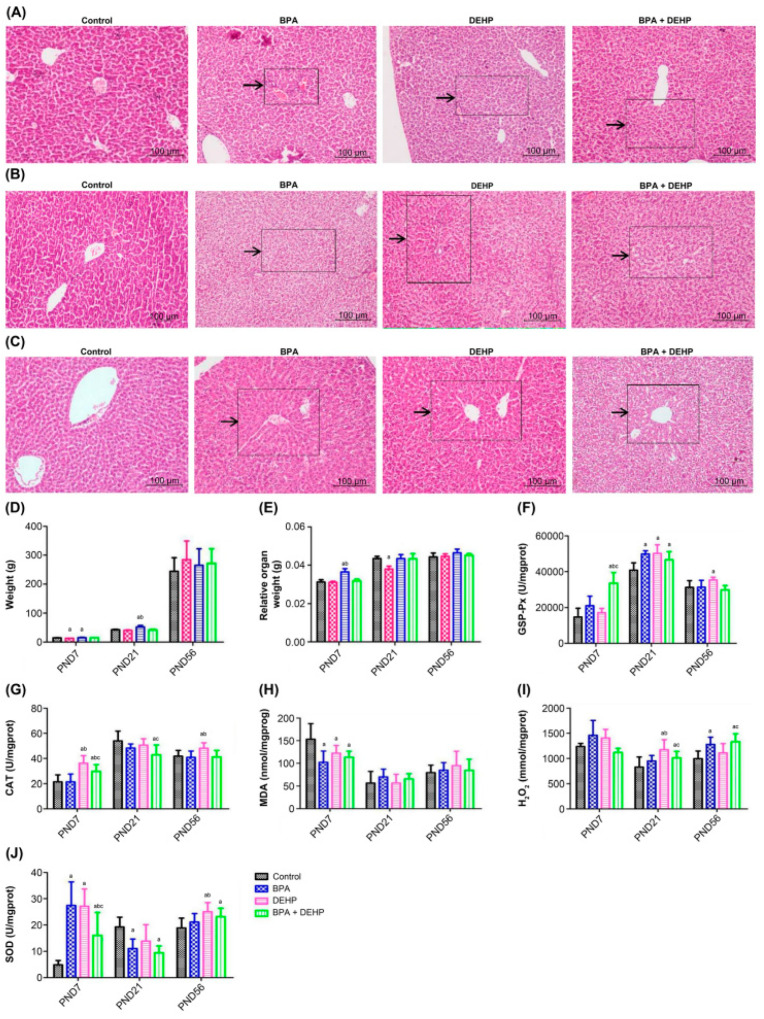
Histopathological changes of PND7, PND21 and PND56 of offspring with haematoxylin and eosin (**A**–**C**). Body weight gain of offspring exposed to BPA, DEHP and BPA+DEHP (**D**). Relative organ weight of offspring exposed to BPA, DEHP and BPA+DEHP (**E**). GSH-PX activity changes of offspring exposed to BPA, DEHP and BPA+DEHP (**F**). CAT activity changes of offspring exposed to BPA, DEHP and BPA+DEHP (**G**). H_2_O_2_ assay of offspring exposed to BPA, DEHP and BPA+DEHP. (**H**). MDA activity changes of offspring exposed to BPA, DEHP, and BPA+DEHP. (**I**). Superoxide dismutase activity of offspring exposed to BPA, DEHP and BPA+DEHP (**J**). Data are presented as means ± SEM. [Note]: “a”: compared with control, “b”: compared with BPA group, “c”: compared with DEHP group, *p* < 0.05, *n* = 6. Scale bar = 1000 μm.

Hepatic histopathological modifications were observed during light microscopic examination, while histological abnormalities were not observed in the control group that presented normal width, cellularity, and morphology of portal spaces. On postnatal day (PND)7, hepatocellular necrosis and fat vacuole degeneration occurred in the DEHP group, and diffuse fat vacuolar degeneration appeared in the BPA+DEHP group (Figure 3A). On PND21, diffuse fat vacuolar degeneration was observed in both the BPA and DEHP groups, with severe lobular cell hypertrophy around the portal area. Severe diffuse fat vacuolar degeneration occurred in the liver of the mixed group (Figure 3B) while on PND56, lobular cell hypertrophy and hepatic sinus dilatation were found in the BPA group. Hepatic fat vacuolar degeneration and lobular cell hypertrophy were found in the DEHP group. Hepatic granuloma, Kupffer cell proliferation, diffuse fat vacuolar degeneration and hepatic cell necrosis were found in the BPA+DEHP group (Figure 3C).

#### 3.3.2. Body Weight Gain (BWG) in Offspring

BWG was significantly lower in BPA and DEHP groups after the first week of exposure. On PND21, reduction in body weight could only be observed in the DEHP group (*p* < 0.05). On PND56, no significant difference was observed in BWG among all groups (*p* > 0.05) (Figure 3D). However, according to the World Health Organization (WHO) guidance document (Pesticide Residues in Food) of the WHO Core Assessment Group on Pesticide Residues, a weight difference above or below the control value by 10% is considered as an adverse change or increase/decrease in weight. Since most of the observed changes in the BWG are beyond the range of *±* 10% according to these criteria, they should be considered as adverse changes.

#### 3.3.3. Relative Organ Weights (ROW) in Offspring

There was a significant augment in relative organ weight in the DEHP group on PND7 (*p* < 0.05). On PND21, the difference between the BPA group and the control group was significantly (*p* < 0.05), while on PND56, there was no significant modification in relative organ weight among all groups (Figure 3E).

#### 3.3.4. Effects of Hepatic Oxidative Stress in Offspring 

In the BPA+DEHP group, there was a significant increase of GSP-Px activity in comparison with the control group on PND7 (*p* < 0.05). There was no significant change in the single substance groups (BPA, DEHP groups), suggesting an additional effect of the examined compounds. On PND21, a significant increase in GSP-Px activity compared to the control group and the DEHP group was observed in the combination group (Figure 3F, *p* < 0.05). In the BPA+DEHP group, there was a significant increase of CAT activity compared to the other groups on PND7 (*p* < 0.05). CAT activity was also increased significantly in the DEHP group compared to the control and the BPA group on PND21. Compared to the control and BPA groups, CAT activity was significantly higher in the DEHP group on PND56 (Figure 3G, *p* < 0.05). Malondialdehyde (MDA) was significantly lower compared to the other experimental groups on PND7 (*p* < 0.05), while there was no significant change on both PND21 and PND56 in all groups (Figure 3H, *p* < 0.05). Moreover, there was a significant increase of the H_2_O_2_ content in the DEHP and BPA groups compared to the control group on PND7 (*p* < 0.05). H_2_O_2_ content was obviously higher in the DEHP group and the combination group on PND21 compared to both control and BPA groups (Figure 3I, *p* < 0.05). A significant increase of the superoxide dismutase (SOD) activity was observed in all evaluated groups on PND7 (*p* < 0.05), whereas an obvious rise in the SOD activity was observed in the BPA and combination groups on PND21 (Figure 3J, *p* < 0.05).

#### 3.3.5. Effects of Hepatic Oxidative Stress in Pregnant Females 

**Figure 4:**
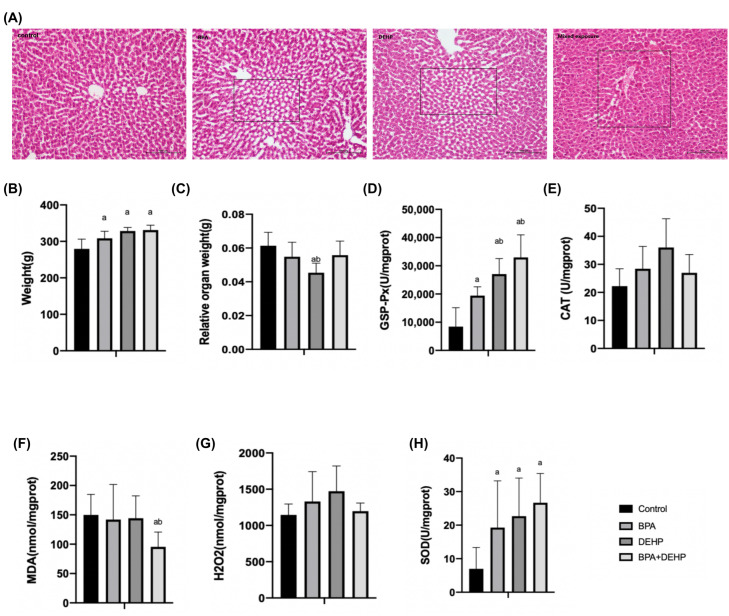
Histopathological changes of pregnant females with haematoxylin and eosin (**A**). Body weight gain of pregnant females exposed to BPA, DEHP and BPA+DEHP (**B**). Relative organ weight of pregnant females exposed to BPA, DEHP and BPA+DEHP (**C**). GSH-PX activity changes of pregnant females exposed to BPA, DEHP and BPA+DEHP (**D**). CAT activity changes of pregnant females exposed to BPA, DEHP and BPA+DEHP (**E**). H_2_O_2_ assay of pregnant females exposed to BPA, DEHP and BPA+DEHP (**F**). MDA assay of pregnant females exposed to BPA, DEHP and BPA+DEHP (**G**). Superoxide dismutase activity of pregnant females exposed to BPA, DEHP and BPA+DEHP (**H**). Data are presented as means ± SEM. [Note]: “a”: compared with control, “b”: compared with BPA group, *p* < 0.05, *n* = 6. Scale bar = 1000 μm.

In the BPA+DEHP group, there was a significant increase of GSP-Px activity in comparison with the other three groups, suggesting an additional effect of the mixture compounds (Figure 4D, *p* < 0.05). No obvious CAT activity or H_2_O_2_ content was changed in all four groups (Figure 4E,G). Malondialdehyde (MDA) was significantly lower compared to the control and BPA groups in the mixture group (Figure 4F, *p* < 0.05). Moreover, a significant increase of the superoxide dismutase (SOD) activity was observed in all evaluated groups (Figure 4H, *p* < 0.05).

### 3.4. Evaluation of the Correlation between DEHP, BPA, and Their Metabolites with Liver Injury 

#### 3.4.1. The Compound Network Pathway BPA, DEHP, and Their Metabolites 

**Figure 5:**
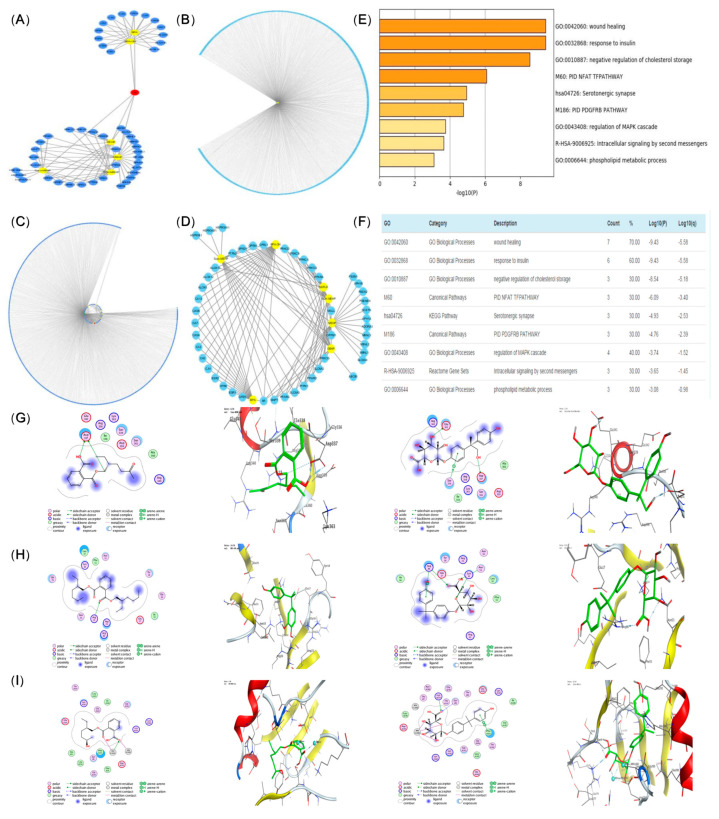
Compound-target-pathway network (blue represents gene targets, yellow represents metabolites, and diseases, red presents co-action targets) (**A**,**B**), Metabolite-targeted interaction network diagram (**C**). Compound-targe-pathway network (blue represents gene targets, yellow represents metabolites and diseases, red presents co-action targets) (**D**), Heat map of target gene set pathway enrichment analysis (**E**), Target gene set pathway enrichment analysis (**F**). Molecular docking of DEHP and BPA prototypes and metabolites to five target proteins: PI3K, AKT, FOXO1 (**G**–**I**).

A total of 54 nodes were obtained from the metabolites-target network, including 48 gene target nodes and 6 metabolite nodes. The interaction revealed that BPA and its metabolites presented the same targets, while DEHP had different targets from its metabolites (Figure 5A,B).

#### 3.4.2. Venn Diagram of Metabolite-Targeted Network and Disease-Targeted Network

A total of 473 nodes were involved in the disease-targeted network, made up of one disease node and 472 gene target nodes. Networks construction was carried out by the merged function of the network visualization software Cytoscape (ver.3.6.0) as follows: (1) Venn diagram of metabolite-targeted network and disease-targeted network (Figure 5C). There were 11 targets in which the evaluated compounds played a role, and the interaction network diagram of metabolites and targets was constructed by taking the targets in the intersection part (Figure 5D). Five biological processes and one KEGG signaling pathway were involved (Figure 5E,F, *p* < 0.05) [21].

### 3.5. Molecular Docking 

In order to verify the accuracy of the network pharmacology prediction results, MOE was performed to simulate the binding of the core metabolites and the five highest core proteins of DEHP and BPA prototypes. Docking scores indicated the degree of docking with BPA and DEHP. The pattern diagram was developed as follows: a higher docking score suggested an improved combination with DEHP and BPA, as presented in Table 4. The higher score docking pattern of PI3K, AKT and FOXO1 proteins with DEHP and BPA prototypes and metabolites molecules are shown in Figure 5G–I. An obvious increase was observed in the combination group compared to the control and BPA groups on PND56. An increase was also observed in the BPA group (Figure 5G–I, *p* < 0.05).

### 3.6. Effects of BPA, DEHP, and BPA+DHEP on the mRNA and Protein Levels of IRS-2, PI3K, AKT, and FOXO1 in Offspring Liver

**Figure 6:**
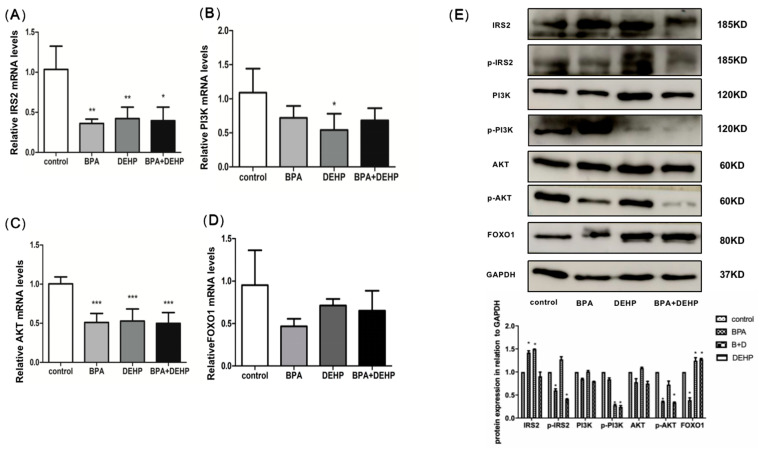
Effect of BPA and DEHP and of their co-exposure-induced mRNA changes in IRS-2 (**A**), PI3K (**B**), AKT (**C**), and FOXO1 (**D**) levels in offspring liver. Effect of BPA group, DEHP group and of their co-exposure induced phosphorylated protein changes in IRS2, PI3K, AKT and FOXO1 levels in offspring liver (**E**). Data are presented as means ± SEM. Data are presented as means ± SEM. [Note]: “*”: *p* < 0.05, “**”: *p* < 0.01, “***”: *p* < 0.001, *n* = 6.

To determine the effects of BPA, DEHP, and their combination as well as the biological pathways they affect, the mRNA levels of proteins with high computer docking scores were examined. Compared with the control, the mRNA levels of insulin receptor substrate 2(IRS-2) and AKT were significantly decreased in all groups (Figure 6A, C, *p* < 0.05). The PI3K mRNA expression level was significantly lower in the DHEP group than in the control group (Figure 6B, *p* < 0.05). In contrast, FOXO1 mRNA levels were not affected significantly by BPA, DEHP, and their combination (Figure 6D). Western blotting was used to assess the abundance of proteins involved in the PI3K/AKT signaling pathway. The protein levels of p-AKT and p-PI3 were reduced significantly by exposure to BPA, DEHP, and their combination (Figure 6E, *p* < 0.05). The protein level of FOXO1 increased significantly after exposure to DEHP and BPA+ DEHP (Figure 6E, *p* < 0.05).

## 4. Discussion

Our study firstly explored gestational and lactational co-exposure to DEHP and BPA on the hepatic function and mechanism in offspring by combining metabolomics, molecular docking, and traditional toxicity assessment methods. EDCs were separated into primary and secondary metabolites by a series of metabolic enzymes to prevent them from being toxic for the organism. It is thus of great significance to explore the toxicology of the metabolic components of exogenous chemicals in biological samples. Epidemiological studies reveal results of EDCs on prenatal growth, thyroid function, obesity, puberty, glucose metabolism, fertility, and carcinogenesis, mainly through epigenetic mechanisms [10]. A simple and rapid precipitation protein method with good reproducibility and recovery was selected for the present study based on China’s standard test method (GB/T21911-2008) [24]. We identified three BPA metabolites and three DEHP metabolites, as well as BPA and DEHP using UPLC-QTOF-MS/MS in gestational rats, and then analyzed the metabolites with a product ion scan (MS/MS) to determine exact mass information. DEHP was stored and exerted a toxic effect either intact or in the forms MEHP, 5OH-MEHP, and 5oxo-MEHP. These findings are in accordance with former studies [25,26,27,28,29,30]. Glucuronidation is thus a common metabolic pathway of DEHP and BPA after synthetical analysis, which provides a new explanation of the effect of the combination of BPA and DEHP.

Capillary electrophoresis-time-of-flight mass spectrometry was used to analyse the metabolomic based on previous reports [31,32,33,34] with detected 19 and 23 peaks in the BPA group and the DEHP group, including changes in lipid metabolism and fatty acid metabolism. PCA analysis and OPLS-DA analysis results also revealed that plasma samples of the control and the infected groups were well separated. Although the metabolic profile of the co-exposure group was different from the single exposure group, metabolomics analysis showed that separation was not satisfied with the co-exposure or the single exposure groups, indicating that there may be no differentiation between the co-exposure group and the single exposure groups in gestational rats. Chromatographic peaks of DEHP, BPA, and their metabolites were not observed in the total sample ion flow chart, which may be related to their weak response based on summaries of previous experience [35,36,37]. Nevertheless, to the best of our knowledge, studies on the subacute toxicity of the mixture of DEHP and BPA are limited, especially considering the effects and mechanism. Our study aim was to compare the effects of co-exposure to DEHP and BPA on different hematological, biochemical, and endocrine function parameters in offspring that were orally administered these chemicals on gestation.

Changes of body weight and organ coefficient in offspring indicating that gestational and lactational exposure to BPA and DEHP could interfere with the normal growth and development of offspring. Pathological findings demonstrated that BPA, DEHP, and their co-exposure during pregnancy could lead to fatty vacuolar degeneration and hepatocyte hypertrophy or necrosis-induced hepatic dysfunction in offspring. We then focused on the underlying mechanisms. NAFLD can be caused by oxidative stress [35] due to an imbalance level between the generation of reactive oxygen species (ROS) and the antioxidant system, including damage to lipids, proteins or DNA in tissues and cells. In the present study, antioxidant enzymes (SOD), glutathione peroxidase (GSH-PX), catalase (CAT), and oxidation products (hydrogen peroxide (H_2_O_2_) and MDA (lipid peroxidation products)) were detected on PND7, 21 and 56. In principle, combined exposure to BPA and DEHP during pregnancy can cause hepatic oxidative stress in offspring according to analysis in other reports [37,38,39,40], and the effects of the combined exposure are different from exposure to the two agents alone, while the interaction mechanism caused by the mixed exposure should be further evaluated. Moreover, these effects on the offspring are likely indirectly driven by direct effects on the pregnant females, rather than direct effects on the offspring. This is also an aspect that warrants further research.

Numerous studies have demonstrated that oxidative stress plays a crucial role in the pathogenesis of NAFLD [41]. The evaluated groups presented lipid metabolism disorder and oxidative damage indicating NAFLD. Furthermore, disease targets were combined with metabolic product targets and it was discovered that 11 gene targets were associated with NAFLD. The targets of the hepatic dysfunction caused by BPA, DEHP, and their metabolites based on NAFLD were then investigated. It was discovered that BPA and its metabolites can target 15 genes, including the estrogen receptor, androgen receptor, carbonic anhydrase and lipoxygenase (ALOX), while DEHP and its metabolites mainly target a total of 34 genes, consisting of protein kinase C (PRKC), AR, single acyl glyceride enzyme (MGLL), non-receptor type of protein tyrosine phosphatase (PTPN), and peroxisome proliferators-activated receptors (PPAR), among others. AR is thus the common target of BPA, DEHP, and their metabolites. Nevertheless, a competitive inhibitory effect may affect the combined toxicity of BPA and DEHP, due to their common target.

KEGG pathway analysis demonstrated the role of BPA and DEHP on hepatic injury and sensitivity to insulin, cholesterol, regulation of MAPK cascade, and phospholipid metabolism, as well as serotonin affecting synaptic pathways. This correlation is also reflected in the previous reports to varying degrees [42,43,44]. To systematically evaluate the mechanisms of gestational and lactational BPA and DEHP co-exposure effect on hepatic function in offspring, we also used computer-aided molecular docking to simulate binding of DEHP and BPA and their metabolites to the top three proteins of interest, namely PI3K, AKT and FOXO1. The glucose metabolism index of perinatal exposure to DEHP and BPA mixture was then evaluated in offspring. The co-exposure group induced changes in PI3K/AKT insulin signal transduction in the liver of offspring. We assume that oxidative stress may occur through the key target genes that coordinately regulate the PI3K/AKT signaling pathway of insulin resistance. IRS is the master switch of the signaling pathway of insulin and the expression of its mRNA was thus examined in this study. IRS-2 mRNA level was obviously reduced in both treated groups, which might be caused by the defective translation of mRNA. PI3K plays an essential role in the organism through insulin signal transduction [45,46]. We found BPA and especially DEHP, along with their co-exposure, significantly diminished mRNA and protein levels of PI3K. Co-exposure to BPA and DEHP may induce specific changes in post-translational modifications of IRS-2, leading to degradation. SH2-domain binding sites were provided by IRS-2 for the regulatory subunit of PI3K, which enable AKT through PIP3 and PDK [47]. AKT protein levels were significantly reduced in all exposure groups. It is thus suggested that DEHP and BPA co-exposure could mediate reduction of IRS-2. The PI3K/AKT signaling pathway plays an essential role in cell growth, proliferation and metabolism, and is also the main downstream molecular pathway of insulin. FOXO1, a transcriptional regulator, is an essential downstream molecule in the insulin signaling pathway and directly regulated by phosphorylation of the upstream PI3K/AKT. We found the effects of co-exposure to BPA and DEHP on FOXO1 are not at the mRNA level, but may be partially due to protein modification or changes in its localization. We randomly selected one rat from a total of six rats (three male and three female) in each group for western blot analysis in our work. While different sex-specific patterns in neonatal rats may have specific effects on signaling pathways, the potential mechanism of DEHP and BPA co-exposures to hepatic function of offspring and the effect of different sex-specific patterns in neonatal rats deserve to be further studied in the further.

## 5. Conclusions

The current study discovered the metabolites of DEHP and BPA in vivo, and compared the plasma metabolism profiles, revealing that the co-exposure group differed from the single exposure group. Evaluation demonstrated that combined or single exposure presented different toxic effects, especially with respect to hepatic dysfunction. Based on network pharmacology and computer-aided molecular docking (MOE) approaches, binding of DEHP, BPA, and their metabolites with the top three proteins of interest was evaluated after analysis of the metabolites. BPA, DEHP, and their combination downregulated the expression of associated genes and proteins in PI3K/AKT pathway. They also upregulated the expression of FOXO1 signaling pathways, causing an increase of glycogen synthesis, reduced glucose uptake, and insulin resistance induction, causing hepatic dysfunction. This is the first study for the hepatic function and mechanism of co-exposure to DEHP and BPA by combining metabolomics, molecular docking, and traditional toxicity assessment methods.

## Figures and Tables

**Table 1:** Number of pregnancies, neonates born, average litter size, gender ratio of offspring born from rat dams following exposure to various treatments.

Group		No. Neonates	Litter Size ($\bar{x}$ ± SD)	Gender (M/M + F)
control	PND7 PND21 PND56	21/21	14.2 ± 1.3	10/21
		18/18	13.5 ± 1.2	8/18
		16/16	14.1 ± 1.0	8/16
BPA	PND7 PND21 PND56	18/20	12.8 ± 0.2	9/18
		17/17	12.2 ± 0.2	8/17
		14/16	12.3 ± 0.3	7/7
DEHP	PND7 PND21 PND56	14/16	11.8 ± 0.7	7/7
		17/17	11.5 ± 1.3	8/9
		14/16	11.7 ± 1.5	7/7
BPA + DEHP	PND7 PND21 PND56	24/28	11.0 ± 0.7	12/12
		22/24	10.6 ± 0.7	10/12
		18/20	10.9 ± 0.3	9/9

**Table 2:** Primer sequence.

Target Gene Orientation	Primer Sequence 5′–3′	Product Size (bp)
IRS2 Forward Primer Reverse Primer	CAGCACCTACGCAAGCATCG GCCCGCCAGCACTTTACTCTTTC	80
PI3K Forward Primer Reverse Primer	AAAACCGGCCAGCTCTTCCA CTTCACAGCACTGGCGGAAC	178
AKT Forward Primer Reverse Primer	ATGGACTTCCGGTCAGGTTCA GCCCTTGCCCAGTAGCTTCA	126
FOXO1 Forward Primer Reverse Primer	TCGGAACGACCTCATGGACG ATGTTGCCTGCTCACTAACTCCT	136

**Table 3:** Gradient elution conditions for HPLC separation.

Time (min)	Mobile Phase A (%)	Mobile Phase B (%)
1 min	5	95
1.01 min	35	65
15 min	90	10
16 min	90	10
16.01 min	5	95
17 min	5	95

**Table 4:** UPLC-Q-TOF/MS analysis results of BPA, DEHP, and their metabolites.

NAME	Molecular	TR (min)	MS (*m/z*)	ppm	Ion Mode	Calculate Mass	Production	Formula
BPA	228.12	2.62	227.11	−4.8	M-H	227.11	133.07/212.09	C_15_H_16_O_2_
BPA-G	404.15	2.61	403.14	−2.7	M-H	403.14	227.11/175.03	C_21_H_24_O_8_
GlcA-BPA-OH	420.14	2.56	419.14	4.8	M-H	419.13	243.10/175.03	C_21_H_24_O_9_
BPA-OH	244.11	2.44	243.10	−3.7	M-H	243.10	ND	C_15_H_16_O_3_
DEHP	390.28	6.95	389.27	−4.1	M-H	389.27	146.97	C_24_H_38_O_4_
MEHP	278.15	3.63	277.15	5.1	M-H	277.14	146.97	C_16_H_22_O_4_
5OH-MEHP	294.15	3.13	293.14	5.5	M-H	293.14	121.03/146.97	C_16_H_22_O_5_
5oxo-MEHP	292.13	3.10	291.13	4.5	M-H	291.12	121.03/146.97	C_16_H_20_O_5_

## Data Availability

Available upon request to the corresponding author.
